# Case 5/2016 - Native Coarctation of the Aortic Arch, Relieved By Percutaneous Treatment in an Adult

**DOI:** 10.5935/abc.20160130

**Published:** 2016-09

**Authors:** Edmar Atik, Raul Arrieta

**Affiliations:** Clínica privada Dr. Edmar Atik, São Paulo, SP - Brazil

**Keywords:** Aortic Disease, Aortic Disease/complications, Cardiac Hypertrophy, Angioplasty, Hypertension

**Clinical data:** the patient had a good clinical course after repair of severe coarctation of the aortic isthmus with end-to-end technique, closure of the interventricular communication at 18 days of life, and relief of moderate subaortic stenosis at 3 years of age. Currently, the patient can tolerate well routine exercise, with no symptoms. At last evaluation, blood pressure in the right arm was 140/70 mmHg and the systolic pressure in the left arm and lower limbs was 90 mmHg, suggesting aortic arch obstruction. Previous evaluations have shown a pressure gradient of 15 mmHg between the upper limbs.

Physical examination: good general health, normal breathing, acyanotic, unequal pulse between the right upper arm and lower extremities. Weight: 65 Kg, height: 165 cm, right upper arm arterial pressure: 140/70 mmHg, left upper arm arterial pressure: 115/80 mmHg, right lower limb arterial pressure: 105/80 mmHg, and heart rate: 82 bpm. The aorta was palpable in the supra-sternal notch, with fremitus and systolic murmur (grade 2).

In the precordium, the apex beat was not palpable and no impulse was detected during systole. Heart sounds were normal, and a harsh, grade 2 systolic murmur was heard in the aortic area and left sternal border with fremitus. The liver was not palpable.

## Complementary tests

**Electrocardiogram** showed sinus rhythm, signs of complete right bundle branch block wit QRS duration of 0.14'', and block of the anterior superior division of the left bundle branch (unchanged since neonatal aortic coarctation repair and interventricular communication). P axis: +20º, QRS axis: +250º, T axis: +35º.

**Chest radiograph** showed normal heart area and myocardial hypertrophy, and normal pulmonary vasculature.

**Echocardiogram** showed normal-sized cardiac chambers, mild myocardial hypertrophy (septum = 14 mm and posterior wall = 11 mm), pressure gradient across the aortic arch = 61 mmHg, and bicuspid aortic valve. Ascending aorta = 29 mm, LA = 32 mm, RV = 26 mm, LV = 48 mm. Septum and left ventricle wall thickness = 10 mm three years ago.

**Figure 1:**
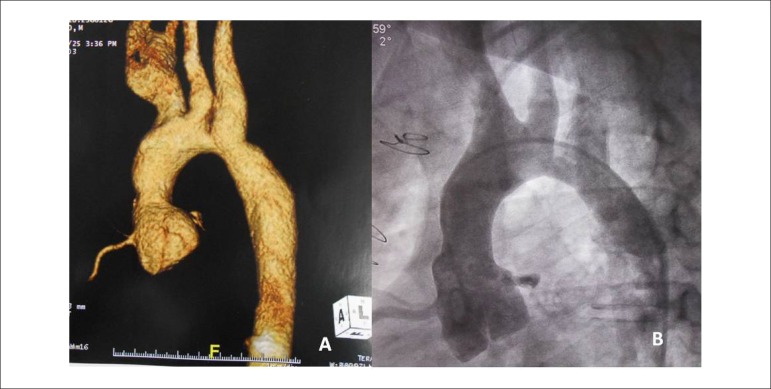
Chest tomography (A) and aortic angiography (B) clearly show the coarctation of the aortic arch after the left carotid artery, featuring coarctation of this region in progression.

**Tomography of thoracic aorta** showed aortic arch obstruction with diameters in the ascending aorta (19 mm), in the arch after left carotid artery (14 mm), in the isthmus after the left subclavian artery at the level where aortic coarctation repair had been performed (24 mm) (Figure 1).

**Ambulatory Blood Pressure Monitoring** showed normal blood pressure in the left arm and blood pressure levels higher than 135/85 mmHg in the right arm in 85% of the time.

**Clinical diagnosis:** Progressive native coarctation of the aortic arch, and previously repaired coarctation of the aortic isthmus.

**Clinical reasoning:** the clinical course was compatible with coarctation of the aortic arch due to the pressure gradient between the upper limbs. The absence of symptoms and the physical tolerance denoted a good dynamic behavior. The absence of pressure gradient between the left upper limb and lower limbs predicted the absence of recoarctation of aortic isthmus. These facts were confirmed by echocardiographic and chest computed tomography images (Figure 1).

**Differential diagnosis:** other diseases accompanied by different levels of aortic obstruction should be considered, such as Kawasaki disease and Takayasu's arteritis, although they are associated with inflammation and occur earlier in life.

**Figure 2:**
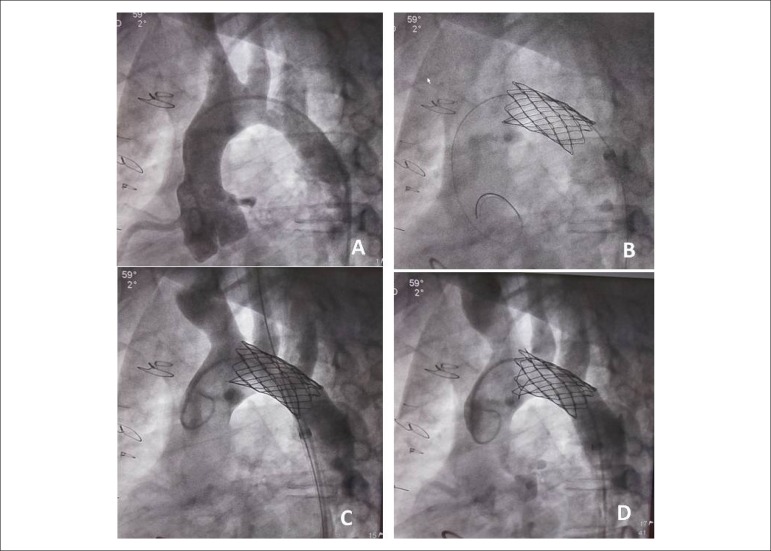
Angioplasty of aortic arch with CP 8Z stent implanted from the brachiocephalic artery to the isthmus. Angiographic image of aortic obstruction after the left carotid artery (A), stent placed during angioplasty (B), stent and angiography of aortic arch (C), and inflated stent in the beginning of the aortic arch.

**Medical management:** given the severity and progression of aortic arch obstruction, systolic artery hypertension and myocardial hypertrophy, surgical correction of the obstruction has been decided on. Placement of a plastic tube between ascending and descending aorta was ruled out and percutaneous treatment was performed. A CP 8Z stent (45 mm) was implanted with a dilation balloon (size 18) from the brachiocephalic artery to the beginning of the descending aorta, with inflation of the stent at the ostium of the left carotid artery (Figure 2). Immediately after stent implantation, the pressure gradient of 20 mmHg decreased and the equalization of pressure was established (96 mmHg). On the next day, blood pressures were 110/70 mmHg in the right arm and 120/80 mmHg in the right dorsalis pedis artery. Heart murmur remained unchanged.

**Comments:** the progressive aortic coarctation after surgical repair at the aortic isthmus is found in 10-20% of cases with long term follow-up. For this reason, aortic coarctation repair performed at early ages has been extended to the aortic arch, using a technique known as "extended end-to-end anastomosis" of the aorta. The diagnosis of coarctation of the aortic arch is easily established in the late stages of the disease provided that blood pressure is systematically measured in all four limbs, as a medical requirement. Narrowing of the aortic arch becomes more evident in the late phase, with no previous parameters for earlier diagnosis of the condition. Therefore, in suspected cases, the extended arch repair for coarctation to becomes the method of choice.

The percutaneous treatment for aortic coarctation, particularly in the isthmus region, has been routinely performed in adult age. Treatment of aortic arch, however, is less frequently performed, despite the favorable clinical course described here and in the literature.

